# Entrapment of Ketorolac Tromethamine in Polymeric Vehicle for Controlled Drug Delivery

**DOI:** 10.4103/0250-474X.59555

**Published:** 2009

**Authors:** S. K. Paliwal, Rajani Chauhan, Veena Sharma, D. K. Majumdar, S. Paliwal

**Affiliations:** Department of Pharmaceutical Science, Faculty of life Sciences, Banasthali Vidyapith-304 022, India; 1 Department of Pharmaceutics, Delhi Institute of Pharmaceutical Sciences and Research, New Delhi-110 017, India; 2 Department of Pharmacy, L. L. R. M. Medical College, Meerut-250 004, India

**Keywords:** Corneal permeation, controlled drug delivery, goat eye, ketorolac tromethamine, polymeric vehicle

## Abstract

The most common method for applying a drug in to the eye is to formulate the drug in the form of an eye drop, but this method is not considered ideal for ocular delivery of drug because of poor bioavailability arising from precorneal loss processes, this loss of drug from the precorneal area is a net effect of drainage, tear secretion and noncorneal absorption. Following the above lead we tried to improve the ocular bioavailability by increasing the corneal contact time and the feasible way was to formulate a drug with mucoadhesive/viscosity imparting agents. The adhesive strength of various polymers on corneal surface was studied with the help of self modified Franz diffusion cell and freshly excised goat/bovine cornea. The polymers hydroxypropylmethylcellulose, carboxymethylcellulose sodium, Eudragit type E/RL/RS, Carbopol ETD 2020 and Carbopol 934 National Formulary were formulated with drug, ketorolac tromethamine. The adhesive strength of polymers on corneal surface and permeation characteristics of drug through cornea were investigated by using above said formulations. Eudragit type E/RL/RS did not show any improvement in mucoadhesion, but the formulations containing Carbopol ETD 2020 and Carbopol 934 national formulary showed good mucoadhesion on corneal surface in the concentration as low as 0.75%. The mucoadhesive strength was also evaluated using the combination of Carbopol acrylates/C 10-30 alkylacrylate with allylpentaerithrital and preservative benzalkonium chloride, which also resulted in good mucoadhesion with improved corneal permeation. Observations made in this study indicate the potentiality of the ophthalmic formulations containing mucoadhesive/viscosity imparting agents.

Ketorolac tromethamine is a non-steroidal antiinflammatory drug (NSAID), received authorization for marketing in the beginning of the nineties for the short-term management of post-operative pains. The tromethamine salt enhances its water solubility and renders the drug fairly appropriate for parenteral administration[1]. Ketorolac is a derivative of pyrrolidine carboxylic acid and is structurally related to tolmetin and zomepirac. Ketorolac, like the other NSAIDs, has analgesic, antipyretic and antiinflammatory activities. These three actions share a common mechanism that is the inhibition of the cyclooxygenase, a critical enzyme involved in the biosynthesis of clinical mediators, like prostaglandins, prostacyclin and thromboxanes[2]. In animal models ketorolac has exhibited an analgesic/antiinflammatory ratio greater than other NSAIDs[3]. For instance, weight by weight ketorolac proved to be 50 times more potent than naproxen in analgesia models but only 3 times more potent in inflammation models[1]. This remarkable dissociation between analgesic and antiinflammatory effects provided the basis for the development of the drug as excellent antiinflammatory and analgesic in clinical settings[4]. Twenty to thirty milligrams (single dose) is effective in patients with moderate to severe postoperative and corneal cancer pain. Ketorolac containing ophthalmic preparations are used frequently to treat itchy eyes caused by allergies and also bring down swelling and redness (inflammation) that can occur after cataract surgery.

During the last two decades, the development of novel mucoadhesive dosage forms has received considerable interest. The progress in this field has recently been summarized in a number of reviews[5–9]. Entrapment of the drug inside a polymer has been an attractive concept. Mucoadhesive polymers may fulfill the desirable features of a prolonged residence time at the site of drug absorption owing to increased contact with the absorbing mucosa, resulting in a steep concentration gradient to favor drug absorption, and localization in specified regions to improve and enhance the bioavailability of the drug[10]. Factors such as cross-linking status, ionic modification, and salt formation can significantly influence the ability of a material to show substantial mucoadhesion in an *in vitro* system[11,12]. Contrary to these factors, it has been postulated that positively charged polymeric hydrogels develop additional molecular forces by electrostatic interaction with negatively charged sugar moieties of the mucosal surface[13]. The mucoadhesive polymer should have a strong hydrogen bond-forming group. For example carboxylate or hydroxyl carry a strong anionic charge and have surface energy properties which favor easy spreading onto the mucus membrane and are nontoxic, non-absorbable, and non interacting with the drug.

In view of above information we attempted to evaluate the mucoadhesive properties of different polymers and *in vitro* trans-corneal permeation of ketorolac and formulation containing mucoadhesive polymer[14].

All the reagents used for present study were of AR grade and instruments used were UV spectrophotometer (Shimadzu, North america), magnetic stirrer (Precision Industries Ltd., India, New Delhi), Digital Balance (Afcoset, Bangalore, India) and a pH meter (Control Dynamics, India).

A modified Franz diffusion cell[15] consisting of 10 ml glass receptor along with a glass donor cell, which also contains an outlet assembly was used for the mucoadhesion permeability studies. A side arm was allowed for sampling of the receptor cell and the receptor chamber was surrounded by a water jacket through which water at 37° was circulated from a thermostat water bath. A Teflon coated magnetic bead was placed at the bottom of the receptor cell to ensure homogencity of the receptor solution. The entire cell was clamped over a magnetic stirrer.The donor compartment represented the conjunctival sac whereas the receiver compartments represented the anterior segment of the eye. Intravenous infusion set was used to control the flow of the saline bicarbonates ringer over cornea to mimic the flow of the tears.

Fresh whole goat and bovine eyeballs were collected within an hour of slaughtering of the animals and the cornea were excised along with 2-4 mm of scleral tissue and washed carefully with cold (4°) normal saline.

The drug (500 mg) was dissolved in 100 ml of water to get 0.5% w/v solution. One millilitre of this solution was diluted with 2 ml of methanol and 22 ml of 0.1N HCl in a volumetric flask, then 1 ml of this solution was further diluted to 25 ml with 0.1N HCl in a volumetric flask to give the final stock solution. From this solution various dilutions were made in order to get different concentration ranging from 0.32 μl/ml to 8 μl/ml and were used for the preparation of standard curve at 313 nm.

Eudragit E/RL/RS, Carbopol 934NF, Carbopol ETD 2020, carboxymethylcellulose sodium (CMC), hydroxypropylmethylcellulose (HPMC) were considered for the present study. Different concentrations of polymers were prepared in 0.8% saline solution and stirred continuously with the help of mechanical stirrer until homogenous dispersions were obtained[16]. After preparation of polymeric solution, ketorolac tromethamine was added and pH of the solution was adjusted to the desired level. Preservative benzalkonium chloride (BKC) (0.01%) and EDTA (0.01%) were also added to the above solution.

The freshly isolated and washed cornea was initially weighed and mounted between the donor and receptor chamber of the diffusion cell by sandwiching the scleral layer between the rims of the two chambers in such a way so that the epithelial surface faced the donor chamber. Bicarbonate ringer was filled in the receptor chamber. It included solution A (100 ml): NaCl-1.24 g, KCl-0.071 g, NaH_2_PO_4_-0.02 g, NaHCO_3_-0.49 g and solution B (100 ml): CaCl_2_ -0.023 g, MgCl_2_ -0.031 g. Equal quantity of solution A and B were mixed to get bicarbonate ringer and allowed to equilibrate the tissue, any air bubble in the receptor chamber was removed by inverting the diffusion cell. The drug polymer solution was placed on the epithelial surface of the cornea through the donor chamber. Bicarbonate ringer solution of pH 7.2 was allowed to flow over the cornea at a flow rate of 12.5 μl/min from an I.V. infusion set. A sample of 1.0 ml was withdrawn from the receptor chamber at 30, 60, 90 and 120 min intervals and diluted with one ml of 0.1N HCl. The samples after dilution were analyzed for ketorolec content by measuring the absorbance at 313 nm in a UV spectrophotometer. The concentrations were determined with the help of a standard curve.

The corneas used for each permeation study[17,18] were evaluated for their hydration level. The wet cornea weight (Ww) was obtained. Each corneal sample was desiccated at 80° to a constant weight (Wd). The percent corneal hydration level (HL %) defined as [1-(Wd/Ww)]×100 were determined for cornea recovered after each permeation study[19].

HPMC and CMC showed mild mucoadhesion at 1% (w/v) concentration. Lower concentration had no mucoadhesion. Eudragit E/RL/RS did not show any mucoadhesion at all. The result indicates that Carbopol ETD 2020 had best mucoadhesion properties on corneal surface in the concentration as low as 0.75 % followed by Carbopol 934NF. Mucoadhesion of Carbopol ETD 2020 and Carbopol 934NF were also studied at different formulation pH. The results indicate that at lower pH (4.5 and 6.0) both polymers have more mucoadhesion then at the pH 7.4. Though the viscosity of the polymeric dispersion was less at lower pH, the probable reason is that at lower pH the carboxylic group of polymer remains protonated and its binding capacity on the corneal surface increases due to hydrogen bonding.

Formulation with Carbopol ETD 2020 showed maximum permeation after 30 min which decreases afterwards, while formulation without Carbopol ETD 2020 showed increasing trend in permeation up to 60 min, but the total quantity of drug permeated was less then obtained with the formulation containing Carbopol ETD 2020. Results are given in Table 1.

**TABLE 1:** MEAN DRUG PERMEATED THROUGH GOAT CORNEA

Time (min)	With Carbopol ETD 2020		Without Carbopol ETD 2020	
		
	Conc. mg/10 ml (n=3)	% Permeation	Conc. mg/10 ml (n=3)	% Permeation
30	0.0692±0.0533	0.1384	0.0062±0.0034	0.0124
60	0.0116±0.0008	0.0232	0.0115±0.0047	0.0230
90	0.0136±0.0089	0.0272	0.0080±0.0009	0.0160
120	0.0220±0.0049	0.0440	0.0096±0.0088	0.0192

Values are mean±SEM in mg/10 ml of n=3 determination in each group. Corneal hydration level is 79.31% (with Carbopol ETD 2020) and 78.41% (without Carbopol ETD 2020)

The permeation of Carbopol 934 NF formulation was however less then obtained with formulation containing Carbopol ETD 2020. Formulation with and without Carbopol 934NF also showed no corneal damage as the hydration level was below 80%. Results are given in Table 2.

**TABLE 2:** MEAN DRUG PERMEATED THROUGH GOAT CORNEA

Time (min)	With Carbopol 934 NF		Without Carbopol 934 NF	
		
	Conc. mg/10 ml (n=3)	% Permeation	Conc. mg/10ml (n=3)	% Permeation
30	0.0143±0.0178	0.0286	0.0043±0.0009	0.0086
60	0.0012±0.0401	0.0025	0.0027±0.0006	0.0054
90	0.0009±0.0011	0.0017	0.0065±0.0013	0.0130
120	0.0009±0.0011	0.0017	0.0056±0.0016	0.0086

Values are expressed in mean ± SEM in mg/10 ml of n = 3 determination in each group. Corneal hydration level is 78.12% (with Carbopol 934 NF), 78.78% (without Carbopol 934 NF)

Addition of BKC to the Carbopol ETD 2020 formulation, increased the permeation after 90 and 120 min. Quantity permeated after 30 min was less than that obtained without BKC up to 120 min. On the other hand addition of EDTA to the formulation containing Carbopol ETD 2020 and BKC decreased the permeation up to 120 min. Quantity permeated after 30 min was less then that obtained without BKC and EDTA. Corneal hydration with BKC and BKC+EDTA was in the physiological range. Results are given in Table 3. Addition of BKC and EDTA to the formulation containing Carbopol 934 NF had no significant influence on permeation. Results are given in Table 4.

**TABLE 3:** MEAN DRUG PERMEATED THROUGH GOAT CORNEA

Time (min)	With Carbopol ETD 2020 + BKC(0.01%)		With Carbopol ETD 2020 + BKC(0.01%) + EDTA(0.01%)	
		
	Conc. mg/10 ml (n=3)	% Permeation	Conc. mg/10 ml (n=3)	% Permeation
30	0.0060±0.0012	0.0120	0.0213±0.0065	0.0426
60	0.0085±0.0021	0.0170	0.0158±0.0050	0.0316
90	0.0158±0.0045	0.0340	0.0181±0.0031	0.0362
120	0.0216±0.0055	0.0432	0.0181±0.0028	0.0362

Values are expressed in mean±SEM in mg/10 ml of n=3 determination in each group. Corneal hydration level is 75.75% (with Carbopol ETD 2020+BKC), 76.47% (with Carbopol ETD 2020+BKC+EDTA) BKC (benzalkonium chloride, 0.01%), EDTA (ethylene diamine tetra acetic acid, 0.01%)

**TABLE 4:** MEAN DRUG PERMEATED THROUGH GOAT CORNEA

Time (min)	With Carbopol 934 NF + BKC(0.01%)		With Carbopol 934 NF + BKC(0.01%) + EDTA(0.01%)	
		
	Conc. mg/10 ml (n=3)	% Permeation	Conc. mg/10 ml (n=3)	% Permeation
30	0.0020±0.0000	0.0040	0.0015±0.0000	0.0030
60	0.0037±0.0011	0.0074	0.0052±0.0188	0.0140
90	0.0042±0.0004	0.0085	0.0022±0.0003	0.0044
120	0.0060±0.0005	0.0120	0.0035±0.0000	0.0070

Values are expressed in mean±SEM in mg/10 ml of n=3 determination in each group. Corneal hydration level is 74.87% (with Carbopol 934 NF+BKC), 75.75 % (with Carbopol 934 NF+BKC+EDTA) BKC (benzalkonium chloride, 0.01%), EDTA (ethylene diamine tetra acetic acid, 0.01%)

Thus ETD 2020 and BKC with and without EDTA were also tried in bovine cornea. Formulation with BKC Carbopol ETD 2020 showed maximum permeation after 60 min while formulation with BKC+EDTA and Carbopol ETD 2020 showed maximum permeation after 90 min. Results are given in Table 5.

**TABLE 5:** MEAN DRUG PERMEATED THROUGH BOVINE CORNEA

Time (min)	With Carbopol ETD 2020 + BKC(0.01%)		With Carbopol ETD 2020 + BKC(0.01%) + EDTA(0.01%)	
		
	Conc. mg/10 ml (n=3)	% Permeation	Conc. mg/10 ml (n=3)	% Permeation
30	0.0060±0.0022	0.0120	0.0027±0.0001	0.0054
60	0.0120±0.0045	0.0240	0.0050±0.0012	0.0100
90	0.0070±0.0015	0.0140	0.0160±0.0015	0.0320
120	0.0120±0.0018	0.0240	0.0050±0.0019	0.0037

Values are expressed in mean±SEM in mg/10 ml of n=3 determination in each group. Corneal hydration level is 72.22% (with Carbopol ETD 2020+BKC), 73.68% (with Carbopol ETD 2020+BKC+EDTA) BKC (benzalkonium chloride, 0.01%), EDTA (ethylene diamine tetra acetic acid,0.01%)

The results of the present studies reveals that addition of mucoadhesive polymer like Carbopol ETD 2020 can significantly improve mucoadhesion and corneal permeation, providing rationale behind inclusion of compatible, mucoadhesive polymers in controlled drug delivery systems for ophthalmic use.
